# Effect of plyometric training and neuromuscular electrical stimulation assisted strength training on muscular, sprint, and functional performances in collegiate male football players

**DOI:** 10.7717/peerj.13588

**Published:** 2022-06-16

**Authors:** Shahnaz Hasan, Gokulakannan Kandasamy, Danah Alyahya, Asma Alonazi, Azfar Jamal, Amir Iqbal, Radhakrishnan Unnikrishnan, Hariraja Muthusamy

**Affiliations:** 1 Physical Therapy Department, College of Applied Medical Sciences, Majmaah University, Al Majmaah, Saudi Arabia; 2 School of Health and Life Sciences, Teesside University, Middlesbrough, United Kingdom; 3 Department of Biology, College of Science, Al-Zulfi-, Majmaah University, Al Majmaah, Riyadh Region, Saudi Arabia; 4 Health and Basic Science Research Centre, Majmaah University, Al Majmaah, Saudi Arabia; 5 Rehabilitation Research Chair, College of Applied Medical Sciences, King Saud University, Riyadh, Saudi Arabia

**Keywords:** Strength, Functional performance, Sprint, Collegiate male football players, Plyometric training, NMES

## Abstract

**Background:**

The study’s objective was to analyze the influence of an 8-week neuromuscular electrical stimulation (NMES) with a plyometric (PT) and strength training (ST) program on muscular, sprint, and functional performances in collegiate male football players.

**Methods:**

Sixty collegiate male football players participated in this randomized controlled trial single-blind study. All the participants were randomly divided into two groups: (1) NMES group (Experimental, *n* = 30) who received NMES assisted ST and (2) sham NMES group (Control, *n* = 30) who received sham NMES assisted ST. In addition, participants from both groups received a PT program; both groups received intervention on three days a week for 8-weeks. The study’s outcomes, such as muscular, sprint, and functional performances, were assessed using a strength test (STN) for quadriceps muscle, sprint test (ST), and single-leg triple hop test (SLTHT), respectively, at baseline pre-intervention and 8-week post-intervention. The interaction between group and time was identified using a mixed design (2 × 2) ANOVA.

**Results:**

Significant difference found across the two time points for the scores of STN: F (1.58) = 5,479.70, *p* < 0.05; SLTHT: F (1.58) = 118.17, *p* < 0.05; and ST: F (1.58) = 201.63, *p* < 0.05. Similarly, the significant differences were found between groups averaged across time for the scores of STN: F (1.58) = 759.62, *p* < 0.05 and ST: F (1.58) = 10.08, *p* < 0.05. In addition, after 8-week of training, Cohen’s d observed between two groups a large to medium treatment’s effect size for the outcome STN (d = 10.84) and ST (d = 1.31). However, a small effect size was observed only for the SLTHT (d = 0.613).

**Conclusions:**

Findings suggest that the effect of PT and ST with either NMES or sham NMES are equally capable of enhancing muscular, sprint, and functional performances in collegiate male football players. However, PT and ST with NMES have shown an advantage over PT and ST with sham NMES in improving muscular performance and sprint performance among the same participants.

## Introduction

Strength and conditioning play an essential role in injury prevention and improving muscle performance (Wilson et al., 2013). In most sports teams or individual sports such as netball, football, and volleyball, muscle strength of the quadriceps is crucial for athletes sporting abilities such as running, sprinting, and jumping (Magalhães et al., 2004). Movements included in this type of training are powerful and fast concentric contractions followed by high-intensity eccentric contractions throughout a high-impact reaction force is proven to enhance performance (da Cunha et al., 2020). Although an athlete’s performance can be influenced by multiple factors (*i.e*., technical, tactical, and physical) (Aán et al., 2021), the main focus of this study was aimed at muscular, physical, and functional performance.

There is a vast literature on different strengthening exercises methods to improve performance and prevent injury in sports (Myer et al., 2011; LaStayo et al., 2003). Neuromuscular electrical stimulation (NMES) is one method that involves the utilization of electrical stimuli to trigger contractions of the muscles. This technique is widely used for strengthening interventions and restoring or preserving the functioning and mass of muscles in sports (Gobbo et al., 2014). Research shows that NMES is more appropriate in improving muscle strength and performance when combined with other training such as plyometric or strength training (Bansal, Zutshi & Munjal, 2020). The quadriceps are the primary muscle group in the lower limb that controls knee movement and increases stability during any dynamic or functional movement (Markovic & Mikulic, 2010). Strength training the quadriceps muscle plays a vital role in improving functional performance in most sports (Antrich & Brewster, 1986). It is essential to keep the quadriceps strong to prevent knee injuries by reducing the shear force in the tibiofemoral joint (Augustsson & Thomee, 2000).

Plyometric training is also known as dynamic or jump training, involves exercises where muscles are required to exert maximum force throughout short intervals with an overall goal of increasing power (Markovic & Mikulic, 2010). Plyometric is seen as a popular training modality, either alone or when combined with other types of training (Markovic & Mikulic, 2010; Antrich & Brewster, 1986; Augustsson & Thomee, 2000; Hasan et al., 2021). The available evidence shows that this type of training has several positive changes, whether this is in athletic performance and muscle functioning abilities (Markovic & Mikulic, 2010). Strength training, also known as resistance training, is distinguished by the deliberate action of muscular contractions against extraneous loads. It is acknowledged as the most convenient approach for strengthening muscles (Wang & Zhang, 2016).

However, there is previous literature on the effects of NMES and strength training on improving muscle strength and physical and functional performance (Gomes da Silva et al., 2018; Paillard, 2018; Basas et al., 2018). Based on our knowledge, there are limited studies and data looking at NMES and strength training effects with more extended intervention (exceeding 4 weeks). In addition, there is a scarcity of data on the effects of sport specific intervention and gender specific sample size. Previous research was more focused on the effect of NMES in post-operative rehabilitation and not on injury prevention or strength training in football players (Gatewood, Tran & Dragoo, 2017). Therefore, the study’s objective was to analyze the influence of an 8-week neuromuscular electrical stimulation (NMES) with a plyometric and strength training program on muscular strength, sprint performance, and functional performance in collegiate male football players. The study hypothesized that the effect of PT and ST with NMES would be more beneficial than PT and ST with sham NMES on muscular, sprint, and functional performances in male collegiate football players.

## Materials and Methods

### Study design

A single-blind two-arm parallel group randomized controlled trial study design was used to determine the beneficial effects of 8-week NMES training program in collegiate male football players.

### Ethical considerations

The study was conducted according to the guidelines of the Declaration of Helsinki and approved by the Chair of Majmaah University for Research Ethics Committee, Saudi Arabia (Ethics number: MUREC-Dec. 15/COM-2020/13-2 dated 15 December 2020).

### Study population

Two hundred colligate male football players were assessed *via* a telephone interview to participate. The team physiotherapist examined one hundred 10 participants who met the inclusion criteria. Young male participants aged 18 and 25 years who participated in football training were included in the study. The participants were excluded with a history of any lower limb surgery, a current injury that affected the lower limb function, cardio-respiratory disease.

A total of sixty participants were divided randomly between the NMES group (experimental group): NMES aided strength training and the sham NMES group (control group): sham NMES aided strength training.

### Procedures

The study was performed between 30 December 2020 and 28 July 2021 at the Rehabilitation center, Majmaah University, Saudi Arabia. Collegiate male football players were recruited from Majmaah and Riyadh sporting clubs and universities. The NMES experimental procedures and potential risks were explained to the participant before signing their informed consent under the Declaration of Helsinki. The College of Applied Medical Science, Ethical Sub-Committee of the Majmaah, Saudi Arabia (Ethics Number: MUREC-Dec. 15/COM-2020/13-2) approved all procedures of this study.

**Figure 1:**
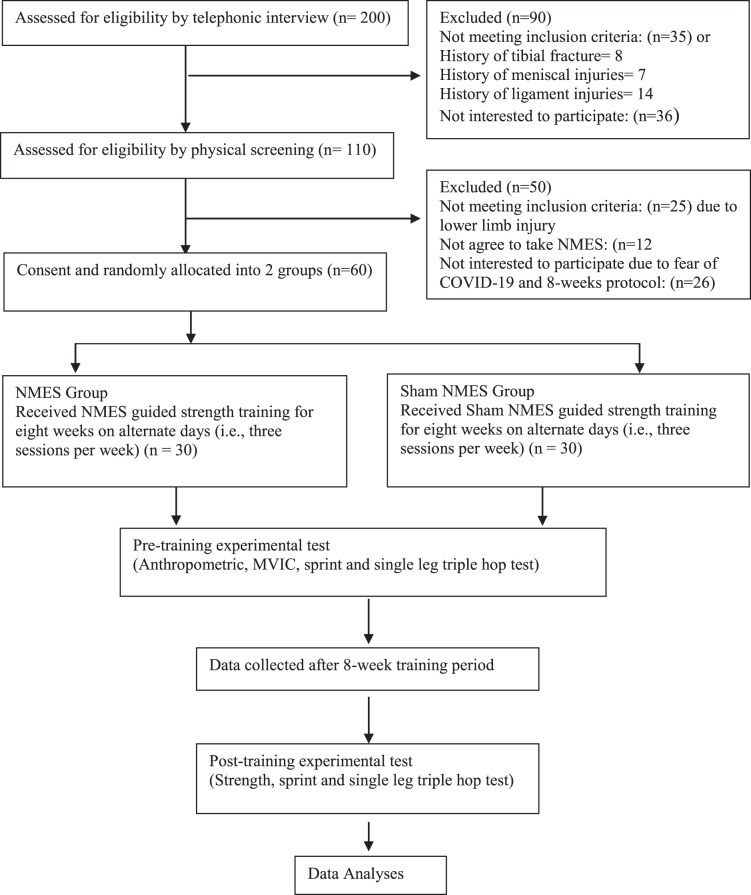
Consolidated Standards of Reporting Trials (CONSORT) diagram showing flow of participants through each stage of a randomized trial.

Conclusively, 60 participants included in this study were randomly divided into two groups: NMES Group (experimental group, *n* = 30) and sham NMES Group (control group, *n* = 30). In addition to plyometric training, both the NMES and sham NMES groups undertook NMES and sham NMES guided strength training, respectively, three sessions a week for 8-weeks. Pre- and post-test readings were taken at baseline and post 8-week of intervention, respectively. The outcome measures for this study were muscular performance *i.e*., maximal voluntary isometric contraction (MVIC) of quadriceps muscle strength measured by ISOMOVE dynamometer (ISO-MANSW-IT Tecno body, Italy; https://www.tecnobody.com/en/products/detail/isomove), the sprint performance test, and the single-leg triple hope test. All the participants who completed the study trial were included for the statistical analysis. A CONSORT flow diagram of the participants is illustrated in Fig. 1 (Schulz, Altman & Moher, 2010).

### Interventions

**Figure 2:**
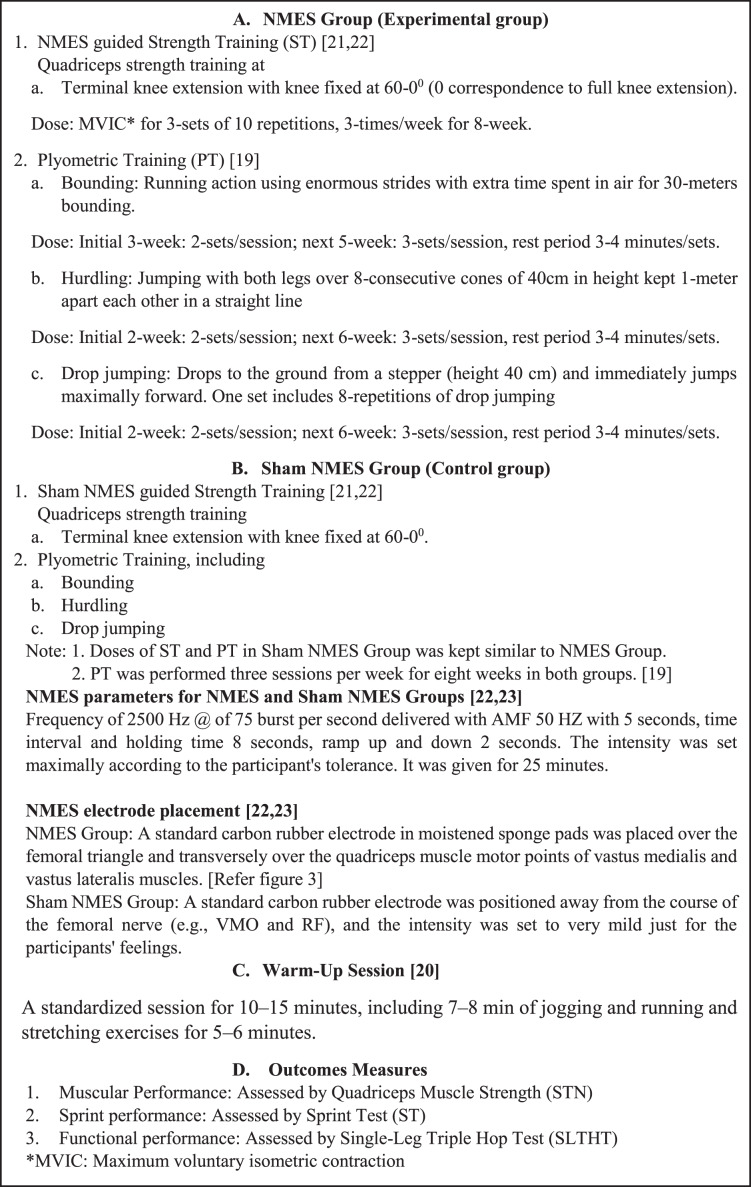
Depicting the details of groups, warm-up activities, interventions including types of exercises, and outcomes measures.

After familiarizing two training and testing sessions, the NMES and sham NMES groups underwent an 8-week training program with three sessions per week. Both groups received the same plyometric training (Tomlinson et al., 2020). In addition, NMES group received the NMES guided strength training while the sham NMES group received the sham NMES guided strength training. Strength training includes terminal knee extension exercises. Plyometric training, including bounding, hurdling, and drop jumping. Before starting any strengthening or plyometric pieces of training, each participant underwent a standardized warm-up session for 10–15 min, which included 7–8 min of jogging and running and stretching exercises for 5–6 min (Silvers-Granelli et al., 2015). Furthermore, Fig. 2 is depicting the details of groups, interventions including types of exercises, and outcomes measures.

**Figure 3:**
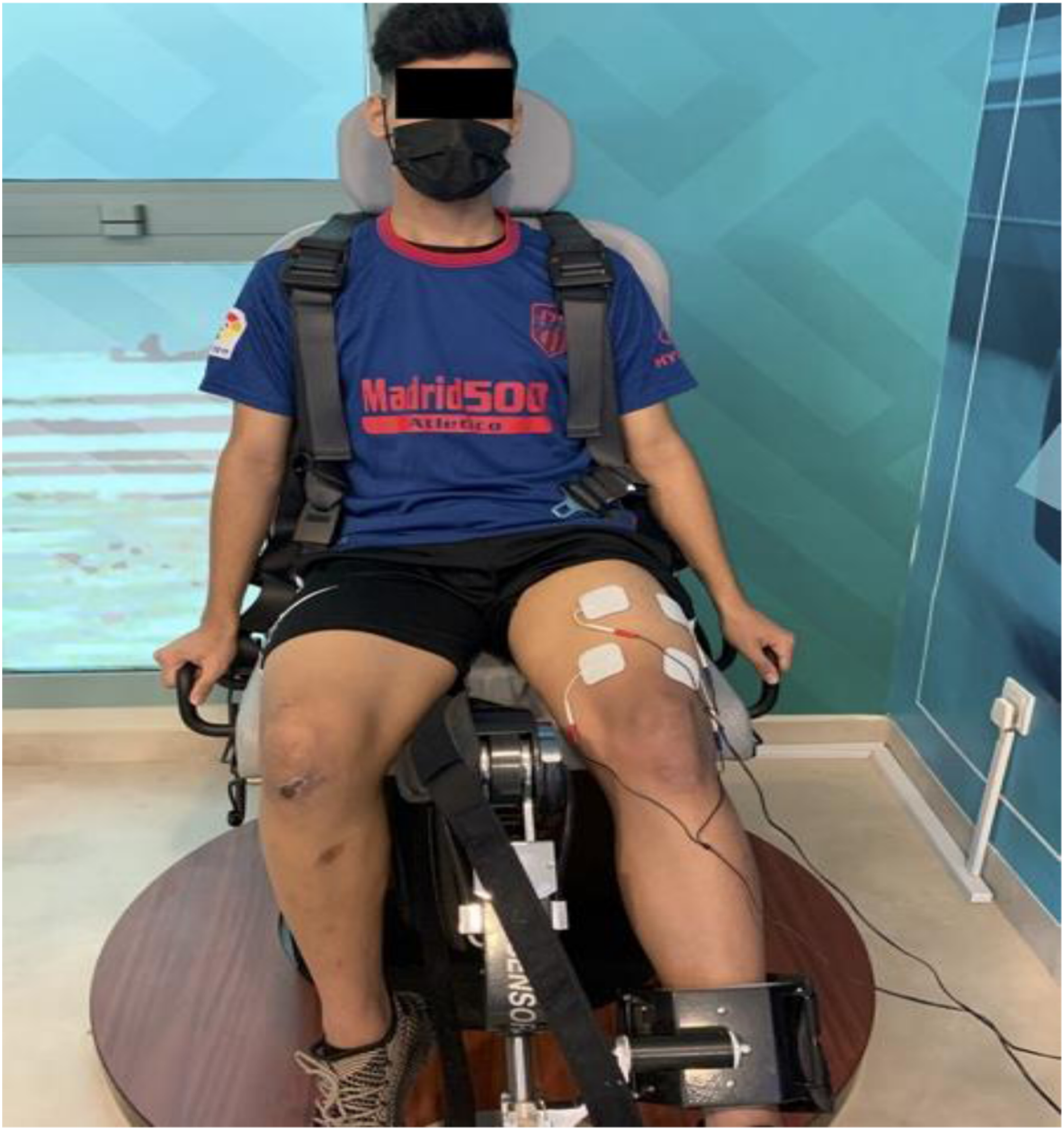
Electrode placement during NMES guided strength training.

Neuromuscular Electrical Nerve Stimulation (Martimbianco et al., 2017; Snyder-Mackler et al., 1995). A NMES guided strength training program was carried out using an electrotherapeutic device (Endomed 982, Enraf Nonius, Rotterdam, The Netherlands), a two-channel medium frequency NME stimulator. In this study, it was applied to stimulate the targeted nerve and muscles, such as the femoral nerve and quadriceps femoris muscles of both limbs. Participants from both groups were instructed to shave the part and wash thoroughly with ethanol to clean the area and reduce skin resistance before applying electrodes over the skin. For the NMES group, a standard carbon rubber electrode in moistened sponge pads was placed over the femoral triangle and transversely over the quadriceps muscle motor points of vastus medialis and vastus lateralis muscles (Fig. 3). Motor points were pointed out as the area that produced the most significant visible muscle contraction when applied electrical stimulation. The electrodes were securely fastened using Velcro straps. The participants were seated on an Isomove device during the stimulation, used for quadriceps strength training with knee fixed at 60 -0-degree angles (0 correspondence to the full extension of the leg) stimulator with a frequency of 2,500 Hz @ of 75 burst per second delivered with AMF 50 HZ with 5 s, time interval and holding time 8 s, ramp up and down 2 s. The intensity was set maximally according to the participant’s tolerance. It was given for 25 min (Snyder-Mackler et al., 1995; Taradaj et al., 2013).

For the sham NMES group, the participants followed the NMES parameters and quadriceps strength training (with knee fixed at 60-0-degree angles) similar to the NMES group. However, in contrast, the placement of electrodes was positioned away from the course of the femoral nerve (*e.g*., VMO and RF), and the intensity was set to very mild just for the participants’ feelings. Each training session lasted for 25 min (Snyder-Mackler et al., 1995; Taradaj et al., 2013).

Terminal knee extension exercises: participants sitting with the knee flexed from 60° to 0° angles on the Isomove device and instructed for maximum voluntary isometric contraction of their quadriceps muscle for three sets of 10 repetitions, three times a week for 8-weeks.

Bounding: This is plyometric training where enormous strides are used in the running action and extra time is spent in the air. The participant performed bounding for 30 m, two sets for the initial 3 weeks, and after three sets of 30 m bounding with a rest period of 3–4 min.

Hurdling: The participant was instructed to jump with both legs over the eight consecutive cones height of 40 cm, kept in a straight line, 1 m apart for plyometric training as hurdling. The participant performed two sets of hurdling over eight cones for the initial 2 weeks. Three sets of hurdling were completed over eight cones for the next 6 weeks. The rest period was 3–4 min between each set of hurdling.

Drop jumping: The participant drops to the ground from a stepper (height 40 cm) and immediately jumps maximally forward. The participant performed two sets of eight repetitions of drop jumping for 2 weeks, and for the next 6 weeks, three sets of eight repetitions of drop jumping with a rest time of 2–3 min between each set were completed (Tomlinson et al., 2020).

For both the NMES and sham NMES groups, plyometric training (bounding, hurdling, and drop jumping) was performed three sessions weekly for 8-weeks (Tomlinson et al., 2020).

### Outcome measures

**Figure 4:**
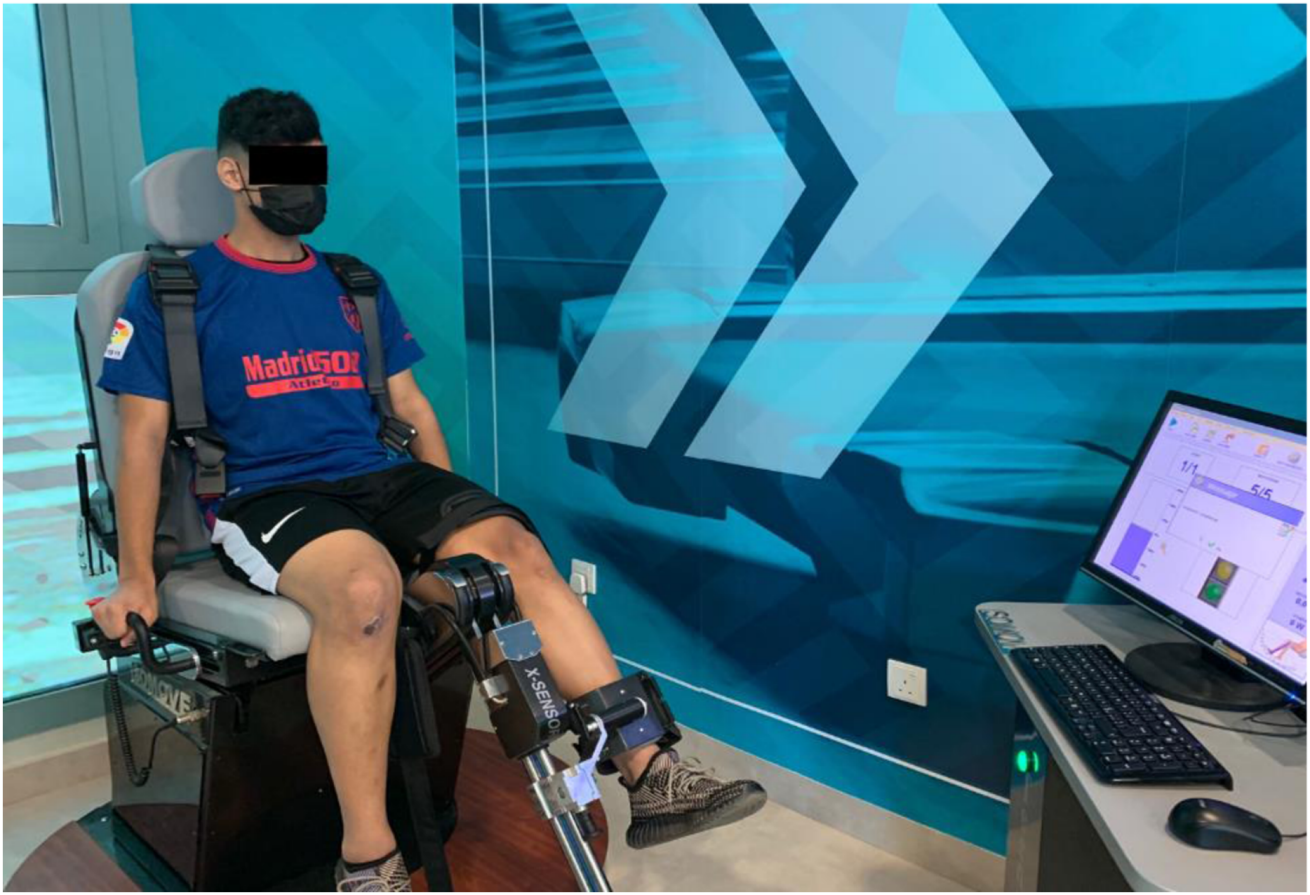
Illustration of maximal voluntary isometric contraction 600 strength (STN) test.

Maximal Voluntary Isometric Contraction Strength (STN) Test: We used an ISOMOVE dynamometer, software version 0.0.1 (ISO-MANSW-IT; Tecnobody, Dalmine (BG), Italy), to assess the maximum peak torque of quadriceps muscles strength dominant leg before strength training and after 8-weeks of training. The reliability of quadriceps strength measurements of the ISOMOVE dynamometer was previously validated (Hasan et al., 2021). Participants completed the warm-up session and were familiarized with the equipment before data collection. Participants were seated with the hip at a 90-degree angle to minimize hip and thigh motion, and straps were applied across the chest, midthighs, and pelvis to avoid displacements during contraction. The shin pad was fixed at 5.1 cm (2 inches), superior to the medial malleolus. The knee angles are set at 60 degrees of flexion, producing the most significant torque output (Alonazi et al., 2021). Verbal instruction was given to keep his/her arm crossed over his/her chest, and verbal encouragement was given to motivate them to attain maximum effort during the 5 s contractions. Each test included three MVICs at 60-degree angles with a 3-min rest between the trials series to eliminate fatigue. The peak torque was directly measured by ISOMOVE software (Fig. 4).

Sprint Test (ST): The sprint test is a reliable (Interclass coefficient correlation = 0.95–0.98) and valid to measure speed performance (Barr et al., 2014; Zabaloy et al., 2021). Participants were instructed to stand with their forward legs placed closer to the starting line, and then on verbal command, they started sprinting with a maximal speed over a 50 m distance. All the performances were recorded by a handheld stopwatch (XINJIE, SW8-2008) times (in seconds) (Hasan et al., 2021; Alonazi et al., 2021; Zafeiridis et al., 2005) when the participant’s foot touched the finishing line. The subsequent two sprint test trials were performed after 5 min recovery period, and the lowest timing of the two scores was considered the pre-test (baseline) scores.

**Figure 5:**
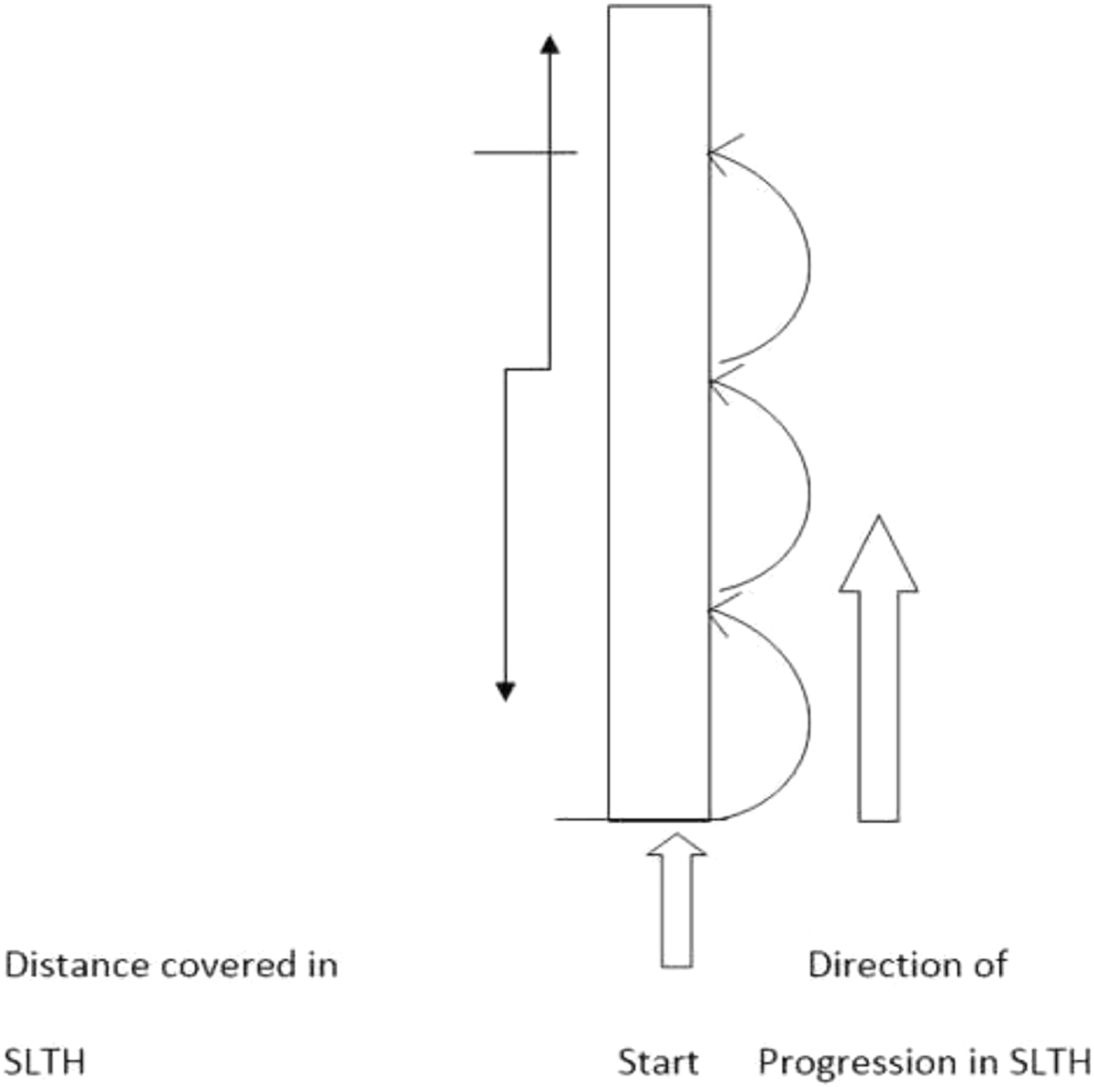
Illustration of single leg triple hop test (SLTHT).

Single-Leg Triple Hop Test (SLTHT): The SLTH test scores were measured from the participant performance, as covered the distance in three hops using a measuring tape. The participants stood on the dominant limb with the toes just behind the starting line and then completed the three consecutive hops on the same limb. The single-leg triple hop test performance measured the distance covered from the starting point to where the back of the participant’s heel hit the ground (please refer to Fig. 5) (Hasan et al., 2021; Alonazi et al., 2021; Kale & Gurol, 2019; Hamilton et al., 2008). They performed three trials with a 3 min’ recovery period. The best of the three scores (*i.e*., the maximum distance covered) was taken as the pre-test (baseline) score.

All the outcomes including MVIC strength (STN), sprint performance (ST), and functional performance (SLTHT) were assessed by only one assessor who was blind to the study. The intra-observer reliability was found to be excellent (95% CI [0.91–0.97]).

### Statistical analysis

A Statistical Programming for Social Studies SPSS software (IBM SPSS Statistics v.26, IBM Corp., Armonk, NY, USA) was used to analyze the outcomes measures. A Shapiro-Wilk test of normality was used for the homogenous distribution of collected data. The main effect of an intervention on the outcome measures across the baseline and 8-week post-intervention (2-time points), between-group (NMES *vs* sham NMES groups), and the interaction between group and time were identified using a mixed design (2 × 2) two-way analysis of variance (ANOVA). Further, the comparison of an intervention effect on the outcome measures within-group across the time points and between-groups at 8-week post-intervention using a Bonferroni’s multiple comparison test. Additionally, the size of an intervention effect on outcomes measures was observed within-group across the time points and between-groups at 8-week post-intervention using a Cohen’s d test. The magnitude of effect sizes in strength training research for untrained participants (who received consistently strength training less than 1-year) is as follows: d value <0.50=trivial effect size, 0.50–1.25=small effect size, 1.26–2.00=medium effect size, and >2.00=large effect size (Rhea, 2004). A relationship among outcomes measures were established using Pearson’s coefficient test. The magnitude of Pearson’s coefficient test *i.e.*, r value between 0.00–0.10, 0.10–0.39, 0.40–0.69, 0.70–0.89, and 0.90–1.00 corresponds to negligible, weak, moderate, strong, and very strong correlation between the variables, respectively (Schober, Boer & Schwarte, 2018). The confidence interval (CI) level was set at 95% for mean, *i.e*., significant level *p* < 0.05.

## Results

Out of 200 telephonic conversations, 110 participants were ready to be examined; out of 110, 25 participants were excluded due to lower limb injury, 12 did not agree to take NMES, and 13 did not agree due to their availability for 8-weeks’ protocol.

The mean scores (95% CI) obtained for the age, height, weight, and BMI of all the participants (*n* = 60) was 22.13 (95% CI [19–25] years), 1.66 (95% CI [1.62–1.70] m), 64.27 (95% CI [55–70] kg), and 23.43 (95% CI [24.40–25.70] kgm^−2^), respectively. A Shapiro-Wilk test reported a homogenous distribution (*p* > 0.05) of descriptive characteristics and outcomes measures among both the groups, except for age (NMES group, *p* = 0.024; sham NMES group, *p* = 0.010), BMI (NMES, *p* = 0.044), and SLTHT (NMES, *p* = 0.013). A group-wise (*n* = 30/group) mean scores for the descriptive characteristic, including age, height, body mass, and BMI of all the participants and outcomes measures, are presented in Table 1.

**Table 1:** Depicting descriptive characteristics of the participants, baseline scores of outcomes measures, and normality test using the Shapiro-Wilk test (95% CI for mean).

Variables	Groups (*n* = 30/group)	Baseline scores (Mean ± SD)	Shapiro-Wilk test of normality				
			Min.	Max.	Statistics	df	*p*-value
Age (years)	NMES	22.20 ± 1.83	19	25	0.918	30	0.024
	Control	22.07 ± 1.80	19	25	0.903	30	0.010
Height (m)	NMES	1.65 ± 0.01	1.63	1.68	0.938	30	0.082
	Control	1.66 ± 0.02	1.62	1.70	0.958	30	0.282
Body mass (Kg)	NMES	63.33 ± 2.99	55	69	0.954	30	0.214
	Control	65.20 ± 2.30	59	70	0.956	30	0.242
BMI (Kg/m2)	NMES	23.23 ± 1.09	20.4	24.9	0.928	30	0.044
	Control	23.63 ± 0.75	21.7	25.7	0.942	30	0.102
STN (Nm-2)	NMES	145.20 ± 3.68	136	151	0.942	30	0.101
	Control	144.93 ± 3.98	135	153	0.975	30	0.685
SLTHT	NMES	501.30 ± 54.50	390	575	0.907	30	0.013
	Control	499.90 ± 51.14	375	586	0.970	30	0.541
ST	NMES	9.19 ± 0.57	7.78	10.37	0.984	30	0.923
	Control	9.23 ± 0.40	8.41	9.96	0.981	30	0.856

**Note:**

Values are mean scores ± standard deviations (SD); BMI, body mass index; NMES, Neuromuscular electrical stimulation group; Control, representing the sham NMES group; Statistics, t-value of t-test; df, Degree of freedom; *p*-value, level of significance; *p* insignificant at >0.05.

Table 2 represents the main effect of the interventions on the outcome measures across the two time points (pre- and post), between the groups, and the interaction between time and group along with the effect size (η). There was a significant difference found across the two time points for the scores of the outcomes STN: F (1.58) = 5,479.70, *p* < 0.05; SLTHT: F (1.58) = 118.17, *p* < 0.05; and ST: F (1.58) = 201.63, *p* < 0.05. Similarly, the significant differences were found between groups averaged across time for the scores of the outcomes STN: F (1.58) = 759.62, *p* < 0.05 and ST: F (1.58) = 10.08, *p* < 0.05. However, a non-significant difference was observed between groups for the scores of the outcomes SLTHT: F (1.58) = 1.53, *p* > 0.05. There was also a significant interaction was observed between time and group for the scores of the outcomes STN: F (1.58) = 1,576.10, *p* < 0.05; SLTHT: F (1.58) = 44.38, *p* < 0.05; and ST: F (1.58) = 24.33, *p* < 0.05.

**Table 2:** The main effect of treatment on the outcomes, within-subject factors across the time (pre and post), between-subject factors between the groups (NMES *vs* Control), and the interaction between groups (2) and time (2) using a mixed design 2 × 2 ANOVA test.

Variables	Outcomes	df1	df2	F-value	*p*-value	η2
Time (2)	STN	1	58	5,479.70	0.001*	0.990
	SLTHT	1	58	118.17	0.001*	0.671
	ST	1	58	201.63	0.001*	0.777
Time * Groups (2 × 2)	STN	1	58	1,576.10	0.001*	0.965
	SLTHT	1	58	44.38	0.001*	0.433
	ST	1	58	24.33	0.001*	0.296
Groups (2)	STN	1	58	759.62	0.001*	0.929
	SLTHT	1	58	1.53	0.221	0.026
	ST	1	58	10.08	0.002*	0.148

**Notes:**

* Significant value if *p* < 0.05.

df, Degree of freedom; η2, Eta Squared where η2 = 0.01 indicates a small effect; η2 = 0.06 indicates a medium effect; η2 = 0.14 indicates a large effect.

Tables 3 and 4 depict pairwise comparisons using Bonferroni multiple comparisons test for the scores of the outcomes within-groups across the two-time points and between-groups at 8 weeks post-intervention, respectively. The findings within-group showed significant differences (*p* < 0.05) for the scores of all outcome measures including STN, ST, and SLTHT across the two-time points of the study (Table 3). However, the between-group analysis demonstrated a significant difference (*p* < 0.05) for the outcomes STN (*p* < 0.001, d = 10.84) and ST (*p* < 0.002, d = 1.31) except for a non-significant difference for the outcome measure SLTHT (*p* > 0.05, d = 0.613) (Table 4). In addition, after 8-week of training, Cohen’s d observed between two groups a large to medium treatment’s effect size for the outcome STN (d = 10.84) and ST (d = 1.31). However, a small effect size was observed only for the SLTHT (d = 0.613).

**Table 3:** Pairwise comparison for the scores of outcomes muscular performance (STN), functional performance (SLTHT), and sprit performance (ST) across two-time points (pre & post) within each group using Bonferroni’s multiple comparison test. Cohen’s d test was applied for measuring effect size between two-time points.

Outcomes	Groups	Pre-intervention	Post-intervention	Time (Pre-Post)	*p*-value	Cohen’s d
STN (∆MD ± SD)	NMES	145.20 ± 3.68	214.67 ± 4.18	−69.47 ± 0.50	0.001*	17.64^^^
	Control	144.93 ± 3.98	165.90 ± 4.80	−20.97 ± 0.82	0.001*	4.76^^^
SLTHT (∆MD ± SD)	NMES	501.30 ± 54.50	540.73 ± 51.78	−39.43 ± 2.72	0.001*	0.74^^^
	Control	499.90 ± 51.14	509.37 ± 50.41	−9.47 ± 0.73	0.004*	0.19
ST (∆MD ± SD)	NMES	9.19 ± 0.58	7.91 ± 0.57	1.28 ± 0.01	0.001*	2.23^^^
	Control	9.23 ± 0.40	8.61 ± 0.50	0.62 ± 0.10	0.001*	1.36^^^

**Notes:**

* Significant value if *p* < 0.05.

^ Large and medium effect size if Cohen’s d value >2.00 and between 1.26–2.00, respectively (Rhea, 2004).

∆MD, Mean differences; SD, Standard Deviation; NMES, Neuromuscular electric stimulation; ∆MD, Mean differences; STN, Strength; SLTHT, Single leg triple hop test; ST, Resisted stride.

**Table 4:** Pairwise comparison of post-test scores (at 8-weeks) for the outcomes muscular performance (STN), functional performance (SLTHT), and sprint test (ST) between groups using Bonferroni multiple comparison test. Cohen’s d test was applied for measuring effect size between two groups.

Outcomes	NMES (Mean ± SD)	Control (Mean ± SD)	NMES *vs* Control (∆MD ± SD)	*p*-value	Cohen’s d
STN	214.67 ± 4.18	165.90 ± 4.80	48.77 ± −0.62	0.001*	10.84^^^
SLTHT	540.73 ± 51.76	509.37 ± 50.41	30.93 ± 0.37	0.221	0.613
ST	7.91 ± 0.57	8.61 ± 0.50	−0.70 ± 0.08	0.002*	1.31^^^

**Notes:**

* Significant value if *p* < 0.05.

^ Large and medium effect size if Cohen’s d value >2.00 and between 1.26-2.00, respectively (Rhea, 2004).

ΔMD, Mean differences; SD, Standard Deviation; NMES, Neuromuscular electric stimulation; STN, Strength test; SLTHT, Single leg triple hop test; ST, sprint test.

In addition, Pearson’s coefficient test revealed a significant (95% CI, *p* < 0.05) but weak to moderate correlation between: STN and SLTHT (r = −0.252, *p* = 0.052), STN and ST (r = −0.540, *p* = 0.001), and ST and SLTHT (r = −0.358, *p* = 0.005) at 8-week post-intervention (Table 5).

**Table 5:** Correlation between strength test (STN), single-leg triple hop test (SLTHT), and sprint test (ST) at post-intervention.

Variables	SLTHT Po (r & *p*-value)	ST Po (r & *p*-value)
STN Po	−0.252 (0.052)*	−0.540 (0.001)*
ST Po	−0.358 (0.005)*	1

**Note:**

* Significant value (2-tailed), if *p* < 0.05.

## Discussion

The present study used NMES and a plyometric training program to assess muscular strength, sprint ability, and functional performance in collegiate male football players. Findings from the 8-week combined program showed improvements in all the above three outcomes. A positive correlation is shown for both experimental and controlled groups in post-intervention variables. Therefore, the results suggest that NMES assisted strength training combined with plyometric training enhances strength and athletic performance in adult male college footballers. The current study adds to existing research, proving that the combination of plyometric training with NMES assisted strength training is an adequate method to improve muscular strength (Alonazi et al., 2021). Similar to the present study, previous studies have shown the effect of NMES on strengthening lower limb muscles in a post-operative population through rehabilitation of knee injuries (Gatewood, Tran & Dragoo, 2017).

For example, in total knee arthroplasty patients, Walls et al. (2010) studied over a similar time frame demonstrated significant improvement in quadriceps strength and functional movements with NMES. In support of our results, a review by Kim et al. (2010) recommended using NMES and exercises together to improve quadriceps strength rather than exercises alone in anterior cruciate ligament (ACL) reconstruction post-operative rehabilitation.

One of the most important reasons for the increased strength is muscle activation potentiation. NMES seems to increase the actin-myosin cross-bridges to calcium, thereby increasing the muscle’s force-generating capacity (da Cunha et al., 2020; Gomes da Silva et al., 2018; Bouguetoch, Martin & Grosprêtre, 2021). Our main findings mainly support the hypothesis in which we found the use of NMES in addition to plyometric training significantly improved strength and physical performance not just immediately but also after 8-weeks of intervention. The muscle fiber type also influences the force-generating capacity of the muscles. Stimulating type II muscle fibers produce a higher specific force than type I fibers. This type of stimulation associated with greater expression of fast-twitch myosin heavy chain isoform through plyometric has proven to increase a muscle’s overall strength and performance (Taradaj et al., 2013; Smith, Hotze & Tate, 2021; Rahmati, Gondin & Malakoutinia, 2021). Although the sham NMES group showed marginal improvements in strength, the magnitude is not the same as the NMES group. Despite the ever-growing scientific research around placebo effects, researchers have continued to present sham procedures with little benefit within clinical research. Brim & Miller (2013) states that the importance of recognizing the extent of the placebo effect from any specific sham-controlled trial is unclear. The placebo effect is well defined for specific sham procedures as it produces a more significant placebo response in more pharmacological research, such as effects on pain rather than enhancing muscular strength, fiber size, and physical performance (Hróbjartsson & Gøtzsche, 2004).

In addition, the perception among researchers and the sports staff is that the NMES to improve strength and performance is more appropriate for the clinical population (who struggle to contract muscles actively) rather than the physically active or athletic population (Veldman et al., 2016). Whereas Thomé et al. (2021) and Gondin, Cozzone & Bendahan (2011) supports the current results as the research states that improvements of up to 40% of athletes sporting performance have been concluded through observation with the use of NMES and the cumulative effect of strong, plyometric training and strengthening protocol (Teixeira et al., 2021a).

The magnitude of effect size in strength training research has been considered relatively higher than in social and psychological research (Rhea, 2004). A very large effect size suggests the practical significance of that particular intervention over the study outcomes. The current study revealed a very high effect size in NMES group (d = 10.84) over sham NMES group for the outcome STN. It advocates the practical significance of NMES, application of NMES as add-on modality is very important in improving the muscle strength, thus, enough reason to consider its application in clinical settings.

Besides many benefits, this study exhibits few limitations for generalizing its finding to some extent. The study was relatively limited to a short duration of the training period (only 8-weeks) in the context for improving strength and physical performance, limited to a very specific study population (*i.e*., collegiate male football players), and did not monitor any external factors, such as additional exercises or physical activities of the collegiate male football players other than their actual intervention that could affect the validity of the study findings (Teixeira et al., 2021b). Therefore, this study cannot be generalized to other populations.

## Conclusions

The research findings suggest that the Plyometric and strength trainings in addition to either NMES or sham NMES for a short-duration training period are equally capable of enhancing the muscular performance, sprint performance, and functional performance of collegiate male football players.

NMES has been proven as a training modality to enhance muscular performance, sprint performance, and functional performance in collegiate male football player; therefore, it might be applied to other similar competitive endurance sports, such as football and netball. Future studies will require more than 8-weeks of training that includes a wider range of endurance athletes, with strict monitoring of external factors that might affect the validity of the findings.

## Supplemental Information

10.7717/peerj.13588/supp-1 Supplemental Information 1 Raw data materials. Master chart

10.7717/peerj.13588/supp-2 Supplemental Information 2 Data analysis. Descriptive and categorical analysis of outcomes

## Additional Information and Declarations

### Data Availability

The following information was supplied regarding data availability: The raw measurements are available in the Supplemental Files.
